# Trabalhadores da saúde expostos a agrotóxicos: um estudo transversal com agentes de combate às endemias do Estado do Rio de Janeiro, Brasil

**DOI:** 10.1590/0102-311XPT045025

**Published:** 2026-04-10

**Authors:** Priscila Jeronimo da Silva Rodrigues Vidal, Liliane Reis Teixeira, Luciana Gomes, Ana Paula das Neves Silva, Marcus Vinicius Corrêa dos Santos, Leandro Vargas Barreto de Carvalho, Ana Cristina Simões Rosa, Ariane Leites Larentis

**Affiliations:** 1 Escola Nacional de Saúde Pública Sergio Arouca, Fundação Oswaldo Cruz, Rio de Janeiro, Brasil.; 2 Escola Politécnica de Saúde Joaquim Venâncio, Fundação Oswaldo Cruz, Rio de Janeiro, Brasil.

**Keywords:** Saúde do Trabalhador, Condições de Trabalho, Exposição Ocupacional, Employee Health, Working Conditions, Occupational Exposure, Salud del Empleado, Condiciones de Trabajo, Exposición Profesional

## Abstract

Nas últimas décadas, o Brasil se tornou um dos maiores consumidores de agrotóxicos do mundo e, além do uso na agricultura, esses compostos são frequentemente usados em ações de saúde pública para combater vetores de doenças endêmicas, por trabalhadores denominados agentes de combate às endemias (ACE), tornando o processo de trabalho nocivo à saúde. O objetivo foi caracterizar a exposição a agrotóxicos, identificar sintomas de intoxicação autorreferidos e a relação com as condições de trabalho e saúde dos ACE do Estado do Rio de Janeiro. Estudo transversal com 606 ACE do Estado do Rio de Janeiro. Foi utilizado um questionário multidimensional autoaplicado de agosto de 2020 a agosto de 2022, e analisado por meio dos testes qui-quadrado e exato de Fisher. Verificou-se a exposição aguda e crônica a múltiplos agrotóxicos, incluindo substâncias carcinogênicas, neurotóxicas e desreguladores endócrinos. Em torno de 51% dos respondentes indicaram dois ou mais sintomas típicos de intoxicação após contato e/ou manipulação. As principais queixas e sintomas foram: dor de cabeça (45,5%), irritação na pele/alergia (38,1%), ardência no nariz e boca, tosse, dificuldades de respirar (33,5%), mal-estar (35,5%), náuseas, vômitos ou diarreia (20%) e fraqueza, tontura ou desmaios (15%). Adicionalmente, foram observados diferentes agravos à saúde dos trabalhadores. Os resultados apontaram para a nocividade das condições e do processo de trabalho dos ACE, com agravos à saúde dos trabalhadores, e a presença de efeitos crônicos. É imprescindível o acompanhamento da saúde desta categoria, e que se priorize práticas para o controle vetorial que não sejam dependentes do uso de agrotóxicos.

## Abstract (en)

In recent decades, Brazil has become one of the largest consumers of pesticides in the world. In addition to use in agriculture, these compounds are often employed in public health actions to combat vectors of endemic diseases by workers denominated vector control workers (VCW), making their work process harmful to health. The aim of the present study was to characterize exposure to pesticides as well as identify self-reported symptoms of poisoning and the relationship with the working and health conditions of VCW. A cross-sectional study was conducted with 606 VCW in the State of Rio de Janeiro, Brazil. A self-administered multidimensional questionnaire was used from August 2020 to August 2022. The results were analyzed using the chi-square test and Fisher’s exact test. Acute and chronic exposure to multiple pesticides was demonstrated, including carcinogenic, neurotoxic, and endocrine-dysregulating substances. Around 51% of the respondents indicated two or more typical symptoms of poisoning after contact and/or handling. The main complaints were headache (45.5%), skin irritation/allergy (38.1%), burning sensation in the nose and mouth, cough, difficulty breathing (33.5%), malaise (35.5%), nausea, vomiting, or diarrhea (20%), and weakness, dizziness, or fainting (15%). Different health problems were also found among the workers. The results demonstrate the harmfulness of the work process of VCW, with health problems for workers and the occurrence of chronic effects. It is essential to monitor the health of these workers and prioritize vector control practices that are not dependent on the use of pesticides.

## Abstract (es)

En las últimas décadas, Brasil se ha convertido en uno de los mayores consumidores de pesticidas del mundo. Además de su uso en la agricultura, estos compuestos se utilizan con frecuencia en acciones de salud pública para combatir los vectores de enfermedades endémicas, por trabajadores denominados agentes de combate a las endemias (ACE), lo que hace que el proceso de trabajo sea perjudicial para la salud. El objetivo fue caracterizar la exposición a plaguicidas, identificar síntomas de intoxicación autoinformados y la relación con las condiciones de trabajo y salud de los ACE del estado de Río de Janeiro. Se realizó un estudio transversal con 606 ACE del estado de Río de Janeiro. Se utilizó un cuestionario multidimensional autoaplicado de agosto de 2020 a agosto de agosto de 2022, que se analizó mediante las pruebas de chi cuadrado y exacta de Fisher. Se verificó la exposición aguda y crónica a múltiples plaguicidas, incluidas sustancias carcinógenas, neurotóxicas y desreguladoras endocrinas. Alrededor del 51% de los encuestados indicaron dos o más síntomas típicos de intoxicación tras el contacto y/o la manipulación. Las principales molestias y síntomas fueron: dolor de cabeza (45,5%); irritación cutánea/alergia (38,1%); ardor en la nariz y la boca, tos, dificultades respiratorias (33,5%); malestar general (35,5%); náuseas, vómitos o diarrea (20%); debilidad, mareos o desmayos (15%). Además, se observaron diferentes problemas de salud entre los trabajadores. Los resultados apuntaron a la nocividad de las condiciones y el proceso de trabajo de los ACE, con problemas de salud para los trabajadores y la presencia de efectos crónicos. Es imprescindible realizar un seguimiento de la salud de esta categoría y dar prioridad a las prácticas de control de vectores que no dependan del uso de plaguicidas.

## Introdução

A história da saúde pública do Brasil está entrelaçada à atuação dos guardas de endemias/agentes de combate às endemias (ACE), cuja origem remonta às brigadas sanitárias de Oswaldo Cruz. Passado mais de um século, e mesmo após organização do Sistema Único de Saúde (SUS), as práticas se mantêm dentro de uma lógica operacional centrada no combate ao vetor, pouco articulada com as especificidades territoriais e às determinações sociais do processo saúde-doença ^1^ ^,^ ^2^.

Não obstante a Política Nacional de Atenção Básica (PNAB) ^3^ tenha ampliado o reconhecimento dos ACE como componente das equipes de atenção primária à saúde (APS), ao propor sua integração com os agentes comunitários de saúde (ACS) e demais profissionais da saúde, essa articulação ocorre de maneira limitada na prática. Nota-se que o trabalho do ACE continua predominantemente voltado para o cumprimento de metas e obtenção de resultados, mantendo o modelo campanhista com uso de agrotóxicos, ainda que com novas denominações. Essa realidade tem resultado na manutenção de condições de trabalho precarizadas e marcada pela exposição crônica a diferentes agrotóxicos, ocasionando processos de trabalho nocivos à saúde ^3^ ^,^ ^4^. 

Nas ações de saúde pública, o emprego de agrotóxicos envolve distintas formas de aplicação, entre as quais: a pulverização em ultra baixo volume (UBV), também conhecida como fumacê, uso de equipamentos costais, nebulizadores, aplicação manual de larvicidas e, mais recentemente, o retorno da borrifação residual intradomiciliar (BRI) ^5^. Todavia, a exposição entre os ACE pode ocorrer em diversas etapas do processo de trabalho, que vão desde o transporte, carga e descarga, até o armazenamento, distribuição, manipulação de equipamentos e preparo da calda/produto, resultando em contato por diferentes vias de exposição ^2^ ^,^ ^4^ ^,^ ^6^. 

Dentre os perigos decorrentes dessa exposição, destaca-se o risco de intoxicação, o qual pode ser potencializado pelas falhas na implementação de medidas de proteção coletiva, pela insuficiência no fornecimento de equipamentos de proteção individual (EPI) adequados, pela deficiência nos treinamentos para o manuseio das substâncias, e do efeito aditivo e/ou sinérgico dos ingredientes ativos manipulados ^7^ ^,^ ^8^ ^,^ ^9^.

Cabe destacar que o Brasil ainda utiliza agrotóxicos proibidos em outros países, como é o caso do Cielo − composto por praletrina e imidacloprida - e também do novalurom, clotianidina, fenitrotiona, alfa-cipermetrina, permetrina e diflubenzurom ^10^ ^,^ ^11^, revelando o contrassenso das políticas de saúde para o controle vetorial e prevenção de doenças endêmicas ^2^ ^,^ ^12^.

As intoxicações por agrotóxicos têm o potencial de causar danos irreversíveis à saúde ^9^ ^,^ ^13^ ^,^ ^14^. Seus efeitos agudos incluem: tontura, irritação nos olhos e pele, dor de cabeça, irritabilidade, náuseas, falta de ar, convulsão, entre outros sintomas. Já os efeitos crônicos podem ser observados mesmo em baixas doses, promovendo danos a diferentes órgãos e sistemas, entre eles: nervoso ^15^ ^,^ ^16^, reprodutivo ^17^, cardiovascular ^18^ e potencializar o risco de câncer ^19^ ^,^ ^20^. Ressalta-se que determinados efeitos relacionados às intoxicações, agudas e crônicas, não são específicos, podendo ser análogos a doenças infecciosas e não-infecciosas, dificultando o diagnóstico e, por consequência, as notificações nos sistemas de informação e da Comunicação de Acidente de Trabalho (CAT) ^7^ ^,^ ^9^ ^,^ ^13^.

Ainda que as campanhas de combate às endemias, com o uso de agrotóxicos, tenham sido implementadas há cerca de 80 anos, com o advento do organoclorado DDT, as pesquisas sobre os efeitos dos agrotóxicos para a saúde dos ACE, apesar de relevantes, ainda são em número reduzido ^6^ ^,^ ^8^ ^,^ ^21^ ^,^ ^22^ ^,^ ^23^ ^,^ ^24^. Essa lacuna torna-se ainda mais evidente quando comparada às pesquisas voltadas aos trabalhadores rurais e outros profissionais da saúde como os ACS. Tal fato aponta para invisibilidade de uma categoria essencial para a história e consolidação da saúde pública do Brasil.

No que tange aos trabalhadores do Estado do Rio de Janeiro, o estudo de Larentis et al. ^6^ analisou certidões de óbito cedidas pelo Núcleo Estadual do Ministério da Saúde no Rio de Janeiro (NERJ) e por familiares, identificando que 75% dos trabalhadores faleceram entre 40 e 59 anos, com idade média de 55 anos, um dado particularmente alarmante quando comparado à expectativa de vida da população brasileira, de 76,4 anos ^25^. As principais causas foram as doenças cardiovasculares (39%) e câncer (15%), ambas reconhecidamente associadas à exposição crônica a agrotóxicos ^9^ ^,^ ^18^ ^,^ ^26^. Esses dados evidenciam o efeito da exposição às substâncias tóxicas sobre a mortalidade precoce da categoria. 

Neste sentido, a fim de contribuir com a Vigilância em Saúde do Trabalhador(a) e visibilizar as condições trabalho e a exposição dos ACE aos agrotóxicos utilizados nas campanhas de saúde pública, o presente estudo teve como objetivo caracterizar a exposição a agrotóxicos, identificar queixas/sintomas de intoxicação autorreferidos e a associação com as condições de trabalho e saúde dos ACE do Estado do Rio de Janeiro.

## Materiais e métodos

### Delineamento, local e população do estudo

Trata-se de um estudo epidemiológico observacional, do tipo transversal, com amostra não probabilística, pois esteve sujeito a disponibilidade de resposta ao questionário, com dois tipos de vínculo institucional: (i) servidores da esfera federal, oriundos da extinta Superintendência de Campanhas de Saúde Pública (Sucam) e da Fundação Nacional de Saúde (Funasa), vinculados ao Ministério da Saúde; e (ii) servidores da esfera municipal, vinculados às secretarias municipais (Prefeituras), com diferentes contratos de trabalho. 

De acordo com dados do Ministério Público do Rio de Janeiro (MPRJ), estima-se que atuem no estado em torno de 7.400 ACE, lotados em sua maioria na Região Metropolitana ^27^. Assim, o presente estudo incluiu trabalhadores que atuam no combate às endemias, de ambos os sexos, lotados nos diferentes municípios do estado e com idade superior a 18 anos. Os critérios de exclusão adotados foram: ser aposentado ou estar afastado do trabalho por tempo indeterminado, ser ACS ou servidores lotados em outros estados.

A pesquisa foi aprovada pelo Comitê de Ética em Pesquisa da Escola Nacional de Saúde Pública Sergio Arouca, Fundação Oswaldo Cruz (CEP/ENSP/FIOCRUZ; sob nº 03323018.4.0000.5240) e integra o projeto multicêntrico que estuda o impacto dos agrotóxicos à saúde dos agentes de combate às endemias e guardas de endemias do Estado do Rio de Janeiro.

### Instrumentos e coleta de dados

O primeiro processo de investigação iniciou-se em 2020, no contexto da pandemia de COVID-19 ^28^ ^,^ ^29^. O estudo foi centrado nas informações referidas, coletadas por meio de um questionário on-line através do Google Forms (https://docs.google.com/forms/u/0/) e divulgado em redes sociais e sítios eletrônicos dos sindicatos da categoria, no período de agosto de 2020 a agosto de 2022.

Os trabalhadores que aceitaram participar da pesquisa concordaram com o Termo de Consentimento Livre e Esclarecido (TCLE) e responderam perguntas sobre o histórico de exposição a agrotóxicos, caracterização do trabalho, condições de saúde, triagem de transtornos mentais comuns (TMC) e avaliação do sono. A linguagem e a construção do questionário foram amplamente discutidas com os representantes sindicais, sobretudo as questões sobre o processo de trabalho e exposição a agrotóxicos, a partir da perspectiva da Comunidade Ampliada de Pesquisa (CAP), compreendendo que a produção de conhecimento envolve a cooperação e confrontação dos saberes técnico-científicos e o saber do trabalhador ^30^.

Para o presente estudo foram investigados os aspectos sociodemográficos (sexo, idade, estado civil, renda familiar, cor/raça e escolaridade), características do processo de trabalho (tipo de vínculo, tempo de serviço, município de lotação, atribuições, tarefas e turno) e questões relacionadas a exposição ocupacional (uso de agrotóxicos, contato, manipulação e/ou aplicação de agrotóxicos, histórico de exposição, formas de aplicação, treinamento, uso e acesso a EPI, tipos de EPI, exposição cutânea, lavagens de uniformes e tipos de substâncias químicas empregadas). 

Em relação ao histórico de exposição a agrotóxicos, averiguou-se o uso de substâncias ao longo de dez anos de trabalho (2010 a 2020) e no período corresponde à realização da pesquisa (2020-2022). A identificação dos produtos utilizados baseou-se em uma lista de agrotóxicos empregados nas campanhas de saúde pública, contendo os respectivos princípios ativos e variações comerciais. A lista foi elaborada a partir de informações obtidas junto ao NERJ e levantamento realizado por Silveira ^31^. Os agrotóxicos mencionados foram posteriormente classificados segundo os grupos químicos aos quais pertencem, de modo a auxiliar a compreensão da variedade e complexidade das exposições às quais os ACE estão submetidos.

Para identificação dos “sintomas de intoxicação”, foi adicionada a seguinte pergunta ao questionário: “Ao utilizar esses agrotóxicos, você apresentou algum desses sintomas?”. Os seguintes sintomas foram avaliados: dor de cabeça, mal-estar, dor no peito, dor no estômago, náuseas, vômitos ou diarreia, ardência no nariz e boca, tosse, dificuldades de respirar, fraqueza, tontura ou desmaios, irritação na pele/alergia, lacrimejamento, irritação ocular, salivação, sudorese excessiva, perda de consciência e convulsão. Considerou-se “possível caso de intoxicação” a ocorrência de duas ou mais queixas/sintomas referidos pelos ACE. O objetivo da categorização foi verificar possíveis associações entre as queixas/sintomas com a exposição a agrotóxicos, o histórico ocupacional, as condições de trabalho e o estado atual de saúde.

No que se refere à condição de saúde, foram investigadas as doenças diagnosticadas (autorreferidas) e problemas de saúde, uso regular de medicamentos, consumo de bebidas alcoólicas, tabagismo, percepção da qualidade do sono e triagem de TMC. A triagem de TMC foi avaliada por meio do *Questionário de Autorrelato* (*Self-Reporting Questionnaire*; SRQ-20). Trata-se de um instrumento com 20 perguntas dicotômicas, validado para o Brasil e com boa consistência interna ^32^. Não é um instrumento de diagnóstico, mas possibilita suspeição de aspectos relacionados com estados de depressão e ansiedade. Para ser considerado caso, utilizou-se o corte ≥ 8 de respostas positivas no SRQ-20, para ambos os sexos ^32^. 

Como critério de análise, foram excluídas as respostas duplicadas do questionário, admitindo-se a última como a mais atual para fins de coleta de dados, o que resultou na análise de 606 questionários, abrangendo trabalhadores de 48 municípios do Estado do Rio de Janeiro.

### Análise estatística

As análises estatísticas foram realizadas no software R versão 4.4.2 (http://www.r-project.org) para obter as estimativas brutas de razões de prevalência e intervalos de 95% de confiança (IC95%), referentes à associação entre as queixas/sintomas típicos de intoxicação e demais variáveis. Para verificar a ocorrência de associações, foram utilizados os testes qui-quadrado de Pearson, exato de Fisher e exato de Fisher com simulação Monte Carlo. Para a medida de tendência central e de dispersão, foram calculadas as frequências, mediana e intervalo interquartil (IQ 25-75), com seus respectivos IC95%. 

## Resultados

### Características sociodemográficas, do processo de trabalho e das condições de saúde 

No Brasil há uma variabilidade de terminologias para designar o cargo de ACE, contudo, no presente estudo, em torno de 83% se autodenominam como agentes de combate às endemias e 10% como guardas de endemias; 77,6% estão vinculados ao serviço público federal, 17,7% são servidores municipais e 4,7% são contratados e/ou celetistas. O tempo médio de trabalho encontrado foi 27,8 anos, com mediana de 32 anos (29-33 anos). Nas Tabelas 1 e 2, encontram-se as características sociodemográficas e de trabalho da população estudada.

**Tabela 1:** Caracterização sociodemográfica de agentes de combate às endemias do Estado do Rio de Janeiro, Brasil, por queixas/sintomas relatados, 2020-2022.

Variáveis	n (%)	Sim		Não	
		n (%)	IC95%	n (%)	IC95%
Sexo					
Homem	413 (68,2)	220 (53,3)	48,3; 58,1	193 (46,7)	41,9; 51,7
Mulher	193 (31,8)	89 (46,1)	39,0; 53,4	104 (53,9)	46,6; 61,0
Faixa etária (anos)					
18-29	26 (4,3)	3 (11,5)	3,0; 31,3	23 (88,5)	68,7; 97,0
30-44	67 (11,1)	19 (28,4)	18,3; 40,9	48 (71,6)	59,1; 81,7
45-59	398 (65,7)	224 (56,3)	51,2; 61,2	174 (43,7)	38,8; 48,8
60-74	115 (19,0)	63 (54,8)	45,3; 64,0	52 (45,2)	36,0; 54,7
Estado civil					
Solteiros	85 (14,0)	30 (35,3)	25,4; 46,5	55 (64,7)	53,5; 74,6
Casados	434 (71,6)	233 (53,7)	48,9; 58,4	201 (46,3)	41,6; 51,1
Separados/Divorciados	74 (12,2)	39 (52,7)	40,8; 64,3	35 (47,3)	35,7; 59,2
Viúvos	13 (2,1)	7 (53,8)	26,1; 79,6	6 (46,2)	20,4; 73,9
Renda familiar (R$)					
1.045-2.090,00	193 (31,8)	100 (51,8)	44,5; 59,0	93 (48,2)	41,0; 55,5
2.091,00-3.135,00	170 (28,1)	105 (61,8)	54,0; 69,0	65 (38,2)	31,0; 46,0
3.136,00-4.181,00	98 (16,2)	55 (56,1)	45,7; 66,0	43 (43,9)	34,0; 54,3
4.181,00-5.225,00	74 (12,2)	33 (44,6)	33,2; 56,6	41 (55,4)	43,4; 66,8
> 5.226,00	71 (11,7)	16 (22,5)	13,8; 34,3	55 (77,5)	65,7; 86,2
Cor/Raça					
Brancos	172 (28,4)	87 (50,6)	42,9; 58,2	85 (49,4)	41,8; 57,1
Pretos e pardos	428 (70,6)	221 (51,6)	46,8; 56,4	207 (48,4)	43,6; 53,2
Amarelos e indígenas	6 (1,0)	1 (16,7)	0,9; 63,5	5 (83,3)	36,5; 99,1
Escolaridade					
Pós-graduação	55 (9,1)	30 (54,5)	40,7; 67,8	25 (45,5)	32,2; 59,3
Ensino Superior completo	107 (17,7)	58 (54,2)	44,3; 63,8	49 (45,8)	36,2; 55,7
Ensino Superior incompleto	82 (13,5)	47 (57,3)	45,9; 68,0	35 (42,7)	32,0; 54,1
Ensino Médio completo	312 (51,5)	146 (46,8)	41,2; 52,5	166 (53,2)	47,5; 58,8
Ensino Fundamental completo	46 (7,6)	26 (56,5)	41,2; 70,8	20 (43,5)	29,2; 58,8
Ensino Fundamental incompleto	4 (0,7)	2 (50,0)	15,0; 85,0	2 (50,0)	15,0; 85,0

IC95: intervalo de 95% de confiança.

Nota: a faixa etária foi distribuída de acordo com a *Pesquisa Nacional de Saúde* (Instituto Brasileiro de Geografia e Estatística); a renda familiar teve como base o salário mínimo vigente no início da pesquisa (R$ 1.045,00).

**Tabela 2:** Análise descritiva da caracterização do trabalho por queixas/sintomas autorreferidos por agentes de combate às endemias. Rio de Janeiro, Brasil, 2020-2022.

Variáveis	n (%)	Sim		Não	
		n (%)	IC95%	n (%)	IC95%
Vínculo					
Federais	470 (77,6)	270 (57,4)	52,8; 61,9	200 (42,6)	38,1; 47,2
Demais vínculos (municipais e celetistas)	136 (22,4)	39 (28,7)	21,4; 37,2	97 (71,3)	62,8; 78,6
Tempo de trabalho (anos)					
1-10	64 (10,6)	12 (18,8)	10,5; 30,8	52 (81,2)	69,2; 89,5
11-20	51 (8,4)	15 (29,4)	17,9; 44,0	36 (70,6)	56,0; 82,1
21-30	63 (10,4)	33 (52,4)	39,5; 65,0	30 (47,6)	35,0; 60,5
≥ 31	428 (70,6)	249 (58,2)	53,3; 62,9	179 (41,8)	37,1; 46,7
Regiões de lotação (n = 600)					
Metropolitana	455 (75,1)	232 (51,0)	46,3; 55,7	223 (49,0)	44,3; 53,7
Norte Fluminense	55 (9,1)	30 (54,5)	40,7; 67,8	25 (45,5)	32,2; 59,3
Costa Verde	16 (2,6)	12 (75,0)	47,4; 91,7	4 (25,0)	8,3; 52,6
Centro-Sul Fluminense	5 (0,8)	2 (40,0)	7,3; 83,0	3 (60,0)	17,0; 92,7
Serrana	8 (1,3)	5 (62,5)	25,9; 89,8	3 (37,5)	10,2; 74,1
Baixadas Litorâneas	32 (5,3)	19 (59,4)	40,8; 75,8	13 (40,6)	24,2; 59,2
Médio Paraíba	16 (2,6)	2 (12,5)	2,2; 39,6	14 (87,5)	60,4; 97,8
Noroeste Fluminense	13 (2,1)	2 (15,4)	2,7; 46,3	11 (84,6)	53,7; 97,3
Atribuições/Tarefas					
Agente de campo					
Sim	429 (70,8)	211 (49,2)	44,4; 54,0	218 (50,8)	46,0; 55,6
Não	177 (29,2)	98 (55,4)	47,7; 62,8	79 (44,6)	37,2; 52,3
Preparo do produto					
Sim	82 (13,5)	62 (75,6)	64,7; 84,1	20 (24,4)	15,9; 35,3
Não	524 (86,5)	247 (47,1)	42,8; 51,5	277 (52,9)	48,5; 57,2
Controle de equipamentos					
Sim	54 (8,9)	40 (74,1)	60,1; 84,6	14 (25,9)	15,4; 39,9
Não	552 (91,1)	269 (48,7)	44,5; 53,0	283 (51,3)	47,0; 55,5
Atividades administrativas					
Sim	77 (12,7)	44 (57,1)	45,4; 68,2	33 (42,9)	31,8; 54,6
Não	529 (87,3)	265 (50,1)	45,8; 54,4	264 (49,9)	45,6; 54,2
Almoxarifado					
Sim	14 (2,3)	10 (71,4)	42,0; 90,4	4 (28,6)	9,6; 58,0
Não	592 (97,7)	299 (50,5)	46,4; 54,6	293 (49,5)	45,4; 53,6
Abastecedor					
Sim	28 (4,6)	20 (71,4)	51,1; 86,0	8 (28,6)	14,0; 48,9
Não	578 (95,4)	289 (50,0)	45,9; 54,1	289 (50,0)	45,9; 54,1
Operador					
Sim	39 (6,4)	30 (76,9)	60,3; 88,3	9 (23,1)	11,7; 39,7
Não	567 (93,6)	279 (49,2)	45,0; 53,4	288 (50,8)	46,6; 55,0
Motorista					
Sim	48 (7,9)	30 (62,5)	47,3; 75,7	18 (37,5)	24,3; 52,7
Não	558 (92,1)	279 (50,0)	45,9; 54,1	279 (50,0)	45,9; 54,1
Mecânico de bomba					
Sim	6 (1,0)	5 (83,3)	36,5; 99,1	1 (16,7)	0,9; 63,5
Não	600 (99)	304 (50,7)	46,6; 54,7	296 (49,3)	45,3; 53,4
Carga e descarga e transporte					
Sim	13 (2,1)	11 (84,6)	53,7; 97,3	2 (15,4)	2,7; 46,3
Não	593 (97,9)	298 (50,3)	46,2; 54,3	295 (49,7)	45,7; 53,8
Combate a roedores e moluscos					
Sim	78 (12,9)	57 (73,1)	61,6; 82,2	21 (26,9)	17,8; 38,4
Não	528 (87,1)	252 (47,7)	43,4; 52,1	276 (52,3)	47,9; 56,6
Educação e comunicação					
Sim	90 (14,9)	50 (55,6)	44,7; 65,9	40 (44,4)	34,1; 55,3
Não	516 (85,1)	259 (50,2)	45,8; 54,6	257 (49,8)	45,4; 54,2
Supervisor/Coordenador					
Sim	102 (16,8)	60 (58,8)	48,6; 68,3	42 (41,2)	31,7; 51,4
Não	504 (83,2)	249 (49,4)	45,0; 53,9	255 (50,6)	46,1; 55,0
Turnos de trabalho					
Parcial	94 (15,5)	36 (38,3)	28,6; 48,9	58 (61,7)	51,1; 71,4
Alternantes	26 (4,3)	12 (46,2)	27,1; 66,3	14 (53,8)	33,7; 72,9
Integral	486 (80,2)	261 (53,7)	49,2; 58,2	225 (46,3)	41,8; 50,8

IC95%: intervalo de 95% de confiança.

A função dos ACE extrapola a tarefa de “matar mosquito” e envolve múltiplas atribuições (Tabela 2), além de outras tarefas não mencionadas em tabela (7,5%), como a vacinação animal, vigilância epidemiológica e ambiental, avaliação de risco biológico e não-biológico, entomologia e reconhecimento geográfico. 

Apesar de desempenharem diversas tarefas, 47,5% não têm acesso a EPI. Os principais itens mencionados foram: luvas (43,7%), botas (31,5%), máscaras (27,4%), macacão (19,3%), óculos de segurança (10,1%) e protetor auricular (8,1%). Verificou-se que quase a totalidade dos ACE (99,3%; IC95%: 98,3; 99,7) lavam os uniformes na própria residência, sendo uma fonte de contaminação não apenas para o trabalhador, mas também para seus familiares.

Mesmo no contexto da pandemia de COVID-19 (2020 a 2022), identificou-se o uso de diversos produtos, inclusive com ação imunotóxica, neurotóxica, carcinogênica, cardiovascular e desregulação endócrina, como a cipermetrina, o diflubenzuron, fenitrotiona e a malationa. Os principais agrotóxicos mencionados e formas de aplicação foram reportados na Tabela 3. 

**Tabela 3:** Análise da exposição ocupacional a agrotóxicos por queixas/sintomas autorreferidos por agentes de combate às endemias. Rio de Janeiro, Brasil, 2020-2022.

Variáveis	n (%)	Sim		Não	
		n (%)	IC95%	n (%)	IC95%
Atividade em contato e/ou manipulação de agrotóxicos					
Não	254 (41,9)	111 (43,7)	37,5; 50,0	143 (56,3)	50,0; 62,5
Sim	352 (58,1)	198 (56,2)	50,9; 61,5	154 (43,8)	38,5; 49,1
Histórico de exposição					
Não	175 (28,9)	63 (36,0)	29,0; 43,6	112 (64,0)	56,4; 71,0
Sim	431 (71,1)	246 (57,1)	52,2; 61,8	185 (42,9)	38,2; 47,8
Aplica agrotóxico atualmente					
Não	312 (51,5)	144 (46,2)	40,5; 51,9	168 (53,8)	48,1; 59,5
Sim	294 (48,5)	165 (56,1)	50,2; 61,8	129 (43,9)	38,2; 49,8
Formas de aplicação					
Ultra baixo volume					
Não	521 (86,0)	254 (48,8)	44,4; 53,1	267 (51,2)	46,9; 55,6
Sim	85 (14,0)	55 (64,7)	53,5; 74,6	30 (35,3)	25,4; 46,5
Bomba de aspersão					
Não	523 (86,3)	246 (47,0)	42,7; 51,4	277 (53,0)	48,6; 57,3
Sim	83 (13,7)	63 (75,9)	65,0; 84,3	20 (24,1)	15,7; 35,0
Bomba costal					
Não	530 (87,5)	258 (48,7)	44,4; 53,0	272 (51,3)	47,0; 55,6
Sim	76 (12,5)	51 (67,1)	55,3; 77,2	25 (32,9)	22,8; 44,7
Nebulizador					
Não	577 (95,2)	289 (50,1)	45,9; 54,2	288 (49,9)	45,8; 54,1
Sim	29 (4,8)	20 (69,0)	49,0; 84,0	9 (31,0)	16,0; 51,0
Larvicida manual					
Não	452 (74,6)	240 (53,1)	48,4;57,8	212 (46,9)	42,2; 51,6
Sim	154 (25,4)	69 (44,8)	36,9; 53,0	85 (55,2)	47,0; 63,1
Treinamento adequado/suficiente					
Sim	179 (29,5)	81 (45,3)	37,9; 52,8	98 (54,7)	47,2; 62,1
Não	265 (43,7)	163 (61,5)	55,3; 67,3	102 (38,5)	32,7; 44,7
Sem treinamento	162 (26,7)	65 (40,1)	32,6; 48,1	97 (59,9)	51,9; 67,4
Utiliza equipamento de proteção individual					
Sim	318 (52,5)	164 (51,6)	45,9; 57,2	154 (48,4)	42,8; 54,1
Não	288 (47,5)	145 (50,3)	44,4; 56,3	143 (49,7)	43,7; 55,6
Exposição cutânea					
Não	198 (32,7)	57 (28,8)	22,7; 35,7	141 (71,2)	64,3; 77,3
Sim	408 (67,3)	252 (61,8)	56,8; 66,5	156 (38,2)	33,5; 43,2
Usa algum agrotóxico *					
Não	215 (35,5)	100 (46,5)	39,7; 53,4	115 (53,5)	46,6; 60,3
Sim	391 (64,5)	209 (53,5)	48,4; 58,5	182 (46,5)	41,5; 51,6
Agrotóxicos utilizados ** (2020-2022)					
*Bacillus thuringiensis*					
Não	528 (87,1)	262 (49,6)	45,3; 54,0	266 (50,4)	46,0; 54,7
Sim	78 (12,9)	47 (60,3)	48,5; 71,0	31 (39,7)	29,0; 51,5
Bendiocarbe					
Não	575 (94,9)	288 (50,1)	45,9; 54,2	287 (49,9)	45,8; 54,1
Sim	31 (5,1)	21 (67,7)	48,5; 82,7	10 (32,3)	17,3; 51,5
Cipermetrina					
Não	539 (88,9)	261 (48,4)	44,1; 52,7	278 (51,6)	47,3; 55,9
Sim	67 (11,1)	48 (71,6)	59,1; 81,7	19 (28,4)	18,3; 40,9
Alfacipermetrina					
Não	577 (95,2)	289 (50,1)	45,9; 54,2	288 (49,9)	45,8; 54,1
Sim	29 (4,8)	20 (69,0)	49,0; 84,0	9 (31,0)	16,0; 51,0
Clotianidina					
Não	579 (95,5)	295 (50,9)	46,8; 55,1	284 (49,1)	44,9; 53,2
Sim	27 (4,5)	14 (51,9)	32,4; 70,8	13 (48,1)	29,2; 67,6
Diflubenzurom					
Não	541 (89,3)	266 (49,2)	44,9; 53,5	275 (50,8)	46,5; 55,1
Sim	65 (10,7)	43 (66,2)	53,3; 77,1	22 (33,8)	22,9; 46,7
Espinosade					
Não	471 (77,7)	243 (51,6)	47,0; 56,2	228 (48,4)	43,8; 53,0
Sim	135 (22,3)	66 (48,9)	40,2; 57,6	69 (51,1)	42,4; 59,8
Fenitrotiona					
Não	568 (93,7)	283 (49,8)	45,6; 54,0	285 (50,2)	46,0; 54,4
Sim	38 (6,3)	26 (68,4)	51,2; 82,0	12 (31,6)	18,0; 48,8
Cielo					
Não	582 (96,0)	295 (50,7)	46,5; 54,8	287 (49,3)	45,2; 53,5
Sim	24 (4,0)	14 (58,3)	36,9; 77,2	10 (41,7)	22,8; 63,1
Malationa					
Não	523 (86,3)	256 (48,9)	44,6; 53,3	267 (51,1)	46,7; 55,4
Sim	83 (13,7)	53 (63,9)	52,5; 73,9	30 (36,1)	26,1; 47,5
Novalurom					
Não	552 (91,1)	278 (50,4)	46,1; 54,6	274 (49,6)	45,4; 53,9
Sim	54 (8,9)	31 (57,4)	43,3; 70,5	23 (42,6)	29,5; 56,7
Piriproxifem					
Não	549 (90,6)	292 (53,2)	48,9; 57,4	257 (46,8)	42,6; 51,1
Sim	57 (9,4)	17 (29,8)	18,8; 43,6	40 (70,2)	56,4; 81,2

Cielo: composto por praletrina e imidacloprida; IC95%: intervalo de 95% de confiança.

* A variável se refere aos trabalhadores que mencionaram uso de alguma substância em seu processo de trabalho.

** Na tabela encontram-se as substâncias mais referidas pelos ACE (n > 20) de um total de 18 substâncias mencionadas.

Além disso, observou-se que, ao longo do tempo (2010-2020), foram utilizados diferentes grupos químicos, caracterizando uma longa exposição, principalmente, aos organosfosforados (48,3%), benzoilureias (42,9%), piretroides (33,3%) e cumarínicos (4,5%). O tempo mediano de aplicação foi 25 anos (15-30 anos). 

No que diz respeito às suas condições de saúde, verificou-se que em torno de 74% referiram alguma doença ou problema de saúde previamente diagnosticado, necessitando uso regular de medicamentos (63,2%). Desses ACE, 62,5% têm doenças crônicas não-transmissíveis (DCNT). Na triagem com SRQ-20, foi observada prevalência de 40,1% para TMC e 5,6% para ideação suicida. Ademais, a qualidade do sono foi percebida como ruim ou muito ruim (63,8%) (Tabela 4).

**Tabela 4:** Análise das variáveis sobre as condições de saúde por sintomas/queixas autorreferidos por agentes de combate às endemias. Rio de Janeiro, Brasil, 2020-2022.

Variáveis	n (%)	Sim		Não	
		n (%)	IC95%	n (%)	IC95%
Doenças/Problemas de saúde					
Sim	450 (74,3)	257 (57,1)	52,4; 61,7	193 (42,9)	38,3; 47,6
Não	156 (25,7)	52 (33,3)	26,1; 41,4	104 (66,7)	58,6; 73,9
Doença cardiovascular/Hipertensão					
Sim	277 (45,7)	158 (57,0)	51,0; 62,9	119 (43,0)	37,1; 49,0
Não	329 (54,3)	151 (45,9)	40,4; 51,4	178 (54,1)	48,6; 59,6
Doenças do aparelho respiratório					
Sim	113 (18,6)	82 (72,6)	63,2; 80,3	31 (27,4)	19,7; 36,8
Não	493 (81,4)	227 (46,0)	41,6; 50,6	266 (54,0)	49,4; 58,4
Diabetes					
Sim	109 (18,0)	62 (56,9)	47,1; 66,2	47 (43,1)	33,8; 52,9
Não	497 (82,0)	247 (49,7)	45,2; 54,2	250 (50,3)	45,8; 54,8
Depressão					
Sim	85 (14,0)	67 (78,8)	68,3; 86,6	18 (21,2)	13,4; 31,7
Não	521 (86,0)	242 (46,4)	42,1; 50,8	279 (53,6)	49,2; 57,9
Alterações no fígado e rins					
Sim	83 (13,7)	59 (71,1)	59,9; 80,3	24 (28,9)	19,7; 40,1
Não	523 (86,3)	250 (47,8)	43,5; 52,2	273 (52,2)	47,8; 56,5
Distúrbios hormonais					
Sim	75 (12,4)	55 (73,3)	61,7; 82,6	20 (26,7)	17,4; 38,3
Não	531 (87,6)	254 (47,8)	43,5; 52,2	277 (52,2)	47,8; 56,5
Tremor essencial					
Sim	56 (9,2)	43 (76,8)	63,3; 86,6	13 (23,2)	13,4; 36,7
Não	550 (90,8)	266 (48,4)	44,1; 52,6	284 (51,6)	47,4; 55,9
Problemas imunológicos					
Sim	39 (6,4)	27 (69,2)	52,3; 82,5	12 (30,8)	17,5; 47,7
Não	567 (93,6)	282 (49,7)	45,5; 53,9	285 (50,3)	46,1; 54,5
Câncer					
Sim	12 (2,0)	9 (75,0)	42,8; 93,3	3 (25,0)	6,7; 57,2
Não	594 (98,0)	300 (50,5)	46,4; 54,6	294 (49,5)	45,4; 53,6
Uso de regular de medicamento					
Sim	383 (63,2)	215 (56,1)	51,0; 61,1	168 (43,9)	38,9; 49,0
Não	223 (36,8)	94 (42,2)	35,6; 48,9	129 (57,8)	51,1; 64,4
Qualidade do sono					
Boa ou muito boa	291 (48)	108 (37,1)	31,6; 43,0	183 (62,9)	57,0 ;68,4
Ruim ou muito ruim	315 (52)	201 (63,8)	58,2; 69,1	114 (36,2)	30,9; 41,8
Transtorno mental comum					
Sim	243 (40,1)	162 (66,7)	60,3; 72,5	81 (33,3)	27,5; 39,7
Não	363 (59,9)	147 (40,5)	35,4; 45,8	216 (59,5)	54,2; 64,6
Ideação suicida					
Sim	34 (5,6)	25 (73,5)	55,3; 86,5	9 (26,5)	13,5; 44,7
Não	572 (94,4)	284 (49,7)	45,5; -53,8	288 (50,3)	46,2; 54,5
Etilismo					
Sim	298 (49,2)	143 (48,0)	42,2; 53,8	155 (52,0)	46,2; 57,8
Não	308 (50,8)	166 (53,9)	48,2; 59,5	142 (46,1)	40,5; 51,8
Fumo					
Nunca fumou	407 (67,2)	195 (47,9)	43,0; 52,9	212 (52,1)	47,1; 57,0
Ex-fumante	126 (20,8)	73 (57,9)	48,8; 66,6	53 (42,1)	33,4; 51,2
Fumante	73 (12,0)	41 (56,2)	44,1; 67,6	32 (43,8)	32,4; 55,9

IC95%: intervalo de 95% de confiança.

### Prevalência de queixas/sintomas de intoxicação e associações com o processo de trabalho, exposição a agrotóxicos e saúde

**Figura 1:**
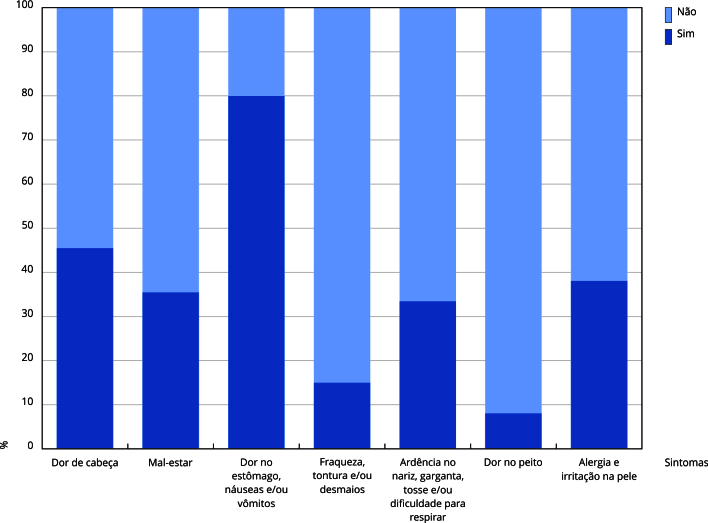
Prevalência dos principais sintomas/queixas autorreferidos por agentes de combate às endemias do Estado do Rio de Janeiro, Brasil (n = 606).

A Figura 1 destaca as principais queixas/sintomas referidos pelos ACE compatíveis com quadros de intoxicação. Analisou-se que 51% (n = 309) indicaram dois sintomas ou mais; 39,3% (n = 238) entre 2-4 sintomas e 11,7% (n = 71) acima de quatro. Ainda que em menor frequência, foram referidos: irritação ocular, lacrimejamento, salivação, sudorese excessiva e lapso de memória (4%). Foram reportados, ainda, episódio de perda de consciência, manifestações compatíveis com quadros psicóticos e crise convulsiva durante a jornada de trabalho, necessitando de internação hospitalar. 

Constatou-se uma forte modulação na prevalência de sintomas em função do vínculo de trabalho. Os servidores federais apresentaram uma prevalência de 57,4% (IC95%: 52,8; 61,9), enquanto os servidores municipais, celetistas/contratados, 28,7% (IC95%: 21,4; 37,2). Ainda que a diferença de magnitude em relação aos servidores federais seja substancial, a estimativa para os demais vínculos é imprecisa devido ao pequeno número de trabalhadores (n = 39).

Entre os servidores com 1-10 anos de atuação, a prevalência foi de 18,8%, atingindo 58,2% entre os servidores com 31 anos ou mais de atuação, onde estão situados os ACE com maior faixa etária e vínculo com serviço público federal, sugerindo os efeitos cumulativos da exposição prolongada (Tabela 2).

A prevalência de queixas/sintomas variou entre as regiões de lotação. Na Região Metropolitana, que compreende a maioria da população (n = 455), a prevalência foi de 51% (IC95%: 46,3; 55,7), como observado na população do estudo. Todavia, observaram-se prevalências marcadamente mais elevadas em regiões com menos participantes, como a Costa Verde (n = 12), onde a prevalência foi de 75%, mas com baixa certeza estatística (IC95%: 47,4; 91,7).

Diversas funções apresentaram prevalências de sintomas notadamente mais elevadas, superando a prevalência geral da amostra (51%). Entre as funções, destacam-se a “carga e descarga e transporte” (84,6%; IC95%: 53,7; 97,3) e “mecânico de bomba” (83,3%; IC95%: 36,5; 99,1). Ainda que estas estimativas sejam imprecisas em decorrência do baixo número de ACE exercendo essas funções (n = 13 e n = 6, respetivamente), as magnitudes são tão elevadas que sugerem a existência de maior nocividade nessas tarefas, necessitando de atenção prioritária. 

Outras tarefas de contato direto com os produtos também apresentaram prevalências altas, porém com estimativas mais estáveis e precisas, como o “preparo do produto” (75,6%), “operador” (76,9%), “controle de equipamentos” (74,1%) e “combate a roedores e moluscos” (73,1%). Em todos estes casos, os limites inferiores dos IC95% permaneceram acima da prevalência observada nos grupos que não exercem essas funções (Tabela 2). Verificou-se que o tipo de turno interferiu na prevalência de sintomas, sendo maior entre ACE que trabalhavam em turno integral (53,7%; IC95%: 49,2; 58,2) (Tabela 2). Trabalhadores com histórico de exposição a agrotóxicos apresentaram prevalência de sintomas de 57,1% (IC95%: 52,2; 61,8), significativamente superior à observada entre aqueles sem esse histórico (36%; IC95%: 29,0; 43,6)

Em relação às formas de aplicação, os ACE que utilizam bomba de aspersão apresentaram maior prevalência de sintomas (75,9%; IC95%: 65,0; 84,3) (Tabela 3). De modo semelhante, os outros métodos de aplicação indicaram valores elevados, como nebulizador (69%), bomba costal (67,1%) e UBV (64,7%) (Tabela 3). Além disso, o tempo médio de aplicação de agrotóxicos foi maior entre os que indicaram dois sintomas ou mais (23,7 anos) do que entre os que não reportaram (20,3 anos).

A Tabela 3 fornece evidências importantes sobre a exposição desses trabalhadores, em especial na via dérmica. A prevalência de sintomas entre os ACE que relataram que “corpo fica exposto a agrotóxicos” foi de 61,8%, mais do que o dobro da prevalência no grupo “não” (28,8%) com IC95% que não se sobrepõem (Tabela 3).

O treinamento revelou um padrão distinto. Os ACE que o consideraram “inadequado”, apresentaram prevalência mais alta (61,5%; IC95%: 55,3; 67,3) do que os que não tiveram treinamento algum (40,1%; IC95%: 32,6; 48,1), sugerindo que o treinamento percebido como inadequado pode estar relacionado a maior nocividade, tarefas de maior exposição ou ainda de adoecimento. Ademais, a prevalência entre os ACE que usam EPI (51,6%; IC95%: 45,9; 57,2) foi semelhante aos não usam/não têm acesso (50,3%; IC95%: 44,4; 56,3). Essa sobreposição quase total dos IC95% indica que o uso de EPI não conferiu proteção efetiva.

A exposição a “piretroides” esteve associada a uma prevalência de 67,3% (IC95%: 60,3; 73,6), comparada a 42,8% (IC95%: 38,0; 47,8), do grupo que “não” utilizou. A exposição aos “organofosforados” mostrou um padrão similar (64,5%; IC95%: 58,7; 69,9) contra 38,3% (IC95%: 33,0; 44,0). Reforçando a plausibilidade biológica dos sintomas e a exposição, foi observada uma clara relação dose-resposta com a quantidade de agrotóxicos utilizados (2010-2020). O grupo com sintomas de intoxicação utilizou, em média, 4,0 tipos de agrotóxicos (IC95%: 3,7; 4,3), enquanto o grupo sem sintomas utilizou 2,6 (IC95%: 2,3; 2,9). A ausência de sobreposição dos IC95% sugere precisão nesta diferença.

Em relação aos aspectos relacionados à saúde dos ACE, as prevalências de sintomas foram marcadamente mais altas entre aqueles que relataram questões relacionadas ao sofrimento psíquico como “depressão” (78,8%; IC95%: 68,3; 86,6), ideação suicida (73,5%; IC95%: 55,3; 86,5), TMC (66,7%; IC95%: 60,3; 72,5) e, ainda, com a qualidade do sono ruim ou muito ruim (63,8%; IC95%: 58,2; 69,1).

Padrões semelhantes, de alta prevalência, foram vistos para ACE com “tremor essencial” (76,8%), “distúrbios hormonais” (73,3%), “doenças do aparelho respiratório” (72,6%) e “alterações no fígado e rins” (71,1%). Todas estas estimativas sugerem um quadro de saúde geral mais debilitado no grupo que referiu queixas/sintomas (Tabela 4). 

Em contraste com as fortes associações vistas, as variáveis “etilismo” e “fumo”, apresentaram prevalências de sintomas muito similares entre os grupos, com intervalos de confiança largamente sobrepostos, sugerindo que não são os principais moduladores do desfecho nesta população (Tabela 4).

Importa relatar que, dos ACE que indicaram tais queixas/sintomas, apenas 25,4% (IC95%: 22,1; 29,0) procuraram atendimento em serviços ou profissionais de saúde. 

## Discussão

O estudo proporcionou informações originais sobre o processo de trabalho dos ACE, caracterizando a exposição a múltiplos agrotóxicos e uma alta prevalência de sintomas indicativos de intoxicação. Ademais, pôs em evidência a nocividade de tarefas, muitas vezes não discutidas, mas que envolvem o contato com substâncias tóxicas, como carga, descarga e transporte, mecânico de bomba, preparo da calda/produto, controle de equipamentos, abastecimento e combate a roedores e moluscos. 

A análise apontou ainda para a ineficiência dos treinamentos e da inadequação dos EPIs, o que sinaliza não apenas as falhas operacionais dessas ações, mas as limitações institucionais relacionadas à qualidade, forma de uso e disponibilidade desses equipamentos. Além disso, o estudo demonstrou uma maior prevalência de queixas entre ACE com relato de exposição cutânea; indicando um aspecto crítico das condições de trabalho.

Esses dados expõem as fragilidades das regulamentações e políticas de saúde, que reconhecem o ACE como ator fundamental nas ações de vigilância, promoção da saúde e na prevenção de agravos coletivos. Contudo, não preconizam a promoção e proteção da saúde do trabalhador, bem como os danos decorrentes dos processos produtivos. Assim sendo, deve-se considerar sua exposição histórica, ainda que os sintomas investigados sejam subjetivos e se assemelhem a outras condições de saúde ^6^ ^,^ ^26^ ^,^ ^33^.

Embora o questionário tenha sido construído em diálogo permanente com os trabalhadores e líderes sindicais, foi observado que vários ACE mencionaram os nomes das substâncias utilizadas no processo de trabalho, mas não reportaram que na sua prática tinham contato ou manipulavam agrotóxicos, o que sugere o desconhecimento sobre o produto que manuseiam e suas nocividades, sobretudo os que manipulam larvicidas (conforme exposto na Tabela 3).

Ademais, muitos ACE atuam concomitantemente no tratamento e bloqueio perifocal manipulando e aplicando agrotóxicos inseticidas e/ou larvicidas, além dos rodenticidas e moluscicidas, frequentemente com formulações de elevada toxicidade, o que pode aumentar o risco de intoxicação. Ressalta-se que os efeitos à saúde podem ser amplificados pela interação sinérgica ou aditiva dos princípios ativos utilizados. ^7^. 

Entre os ACE analisados, observou-se que a ocorrência de doenças crônicas não transmissíveis (DCNT) foi superior (62,5%) à registrada na população geral do Estado do Rio de Janeiro (42,1%; IC95%: 39,9; 44,4) ^34^. Ainda que seja esperada nas faixas etárias mais avançadas maior prevalência de DCNT, esse fato pode mascarar os efeitos da exposição crônica e da complexa relação entre o trabalho e a saúde. Neste sentido, reforça-se a importância de monitoramento ativo e o posicionamento crítico na Vigilância da Saúde do Trabalhador(a).

As exposições crônicas a agrotóxicos, mesmo em baixas doses, são bem documentadas na literatura. Entre os efeitos mais recorrentes, destacam-se: as alterações no sistema imunológico, em órgãos como olhos, pele, rins e fígado ^35^, desregulação endócrina, no sistema reprodutivo e câncer ^11^ ^,^ ^15^ ^,^ ^17^ ^,^ ^20^. A literatura indica ainda a associação entre a exposição ocupacional e doenças cardiovasculares ^15^ ^,^ ^18^, com evidências específicas para ingredientes ativos que foram utilizados pelos ACE, como a fenitrotiona, malationa e deltametrina. Ademais, estudos descrevem efeitos neurológicos e na saúde mental, incluindo disfunções cognitivas, alterações no padrão e qualidade do sono, depressão e ideação suicida ^14^ ^,^ ^15^ ^,^ ^16^.

Essas evidências dialogam diretamente com as condições observadas entre os ACE avaliados, nos quais foram identificados registros dos mesmos agravos descritos na literatura, reforçando a compatibilidade entre os padrões esperados de toxicidade e o perfil de morbidades presente na população estudada.

Ainda no que se refere à saúde mental, Bastos et al. ^24^ identificaram que os transtornos mentais foram as principais causas de afastamento do trabalho entre ACE do Ceará, representando uma parte significativa da história ocupacional vivida com morbidade. De modo convergente, Maturino et al. ^36^ identificaram maior prevalência de TMC entre ACE/ACS, em um estudo sobre trabalhadores da saúde considerados “invisibilizados” e Vidal et al. ^29^, alta prevalência (43%) de TMC em um grupo de ACE do Estado do Rio de Janeiro.

Embora a relação entre o adoecimento psíquico e a exposição a agrotóxicos apresente uma relação bem mais complexa de ser determinada, diante das evidências dos efeitos da exposição à saúde, nos protocolos de triagem de populações cronicamente expostas, preconiza-se a investigação e o monitoramento de efeitos à saúde mental ^37^. Nesse sentido, na atualização da lista de doenças e agravos relacionados ao trabalho, em 2023 ^38^, o Ministério da Saúde incluiu os episódios e transtornos depressivos, e doenças neurológicas, como o tremor, na avaliação da exposição ocupacional a agrotóxicos. 

Em relação aos efeitos neurológicos, Azevedo & Mayer ^23^ identificaram associação significativa entre o tempo de exposição e o desenvolvimento do tremor essencial. Os ACE com mais de 16 anos de exposição tiveram 4,6 vezes mais chance de desenvolver o tremor em comparação àqueles não expostos ocupacionalmente. Tal condição acarreta impactos diretos na qualidade de vida e na saúde mental dos trabalhadores, evidenciando a importância de se considerar os efeitos neurotóxicos da exposição crônica a agrotóxicos.

O estudo de Magalhães & Caldas ^39^, com trabalhadores do Distrito Federal expostos a substâncias químicas, constatou que os organofosforados foram os principais causadores de intoxicação entre agentes de endemias e trabalhadores rurais, destacando ainda o uso de temefós, metamidofós (classificado como extremamente tóxico) e rodenticidas não especificados. Os sintomas mais frequentes incluíam: cefaleia, coceira, náuseas, irritabilidade, além de tremores e depressão, corroborando os dados desse estudo.

De modo semelhante ao que ocorre com a população rural, entre os principais entraves para investigação e confirmação dos casos suspeitos de intoxicação encontram-se a dificuldade dos profissionais de saúde em estabelecerem a relação dos sintomas apresentados com o processo de trabalho, a ausência de registros nos sistemas de informação e a carência de notificações de acidentes de trabalho, sobretudo no que se refere às exposições ocupacionais e aos efeitos crônicos ^7^ ^,^ ^9^ ^,^ ^17^ ^,^ ^26^. 

Em 2019, o Sistema de Informação de Agravos de Notificação (SINAN) registrou 8.412 casos de intoxicações por agrotóxicos, em sua maioria intoxicações agudas ^17^. Observa-se que a comunicação dos casos suspeitos ou prováveis envolve diferentes desafios, como a falta de formação dos profissionais em todo o processo (conferência, digitação e consolidação dos dados), grande demanda dos serviços de saúde e baixa procura dos trabalhadores às unidades de saúde após o surgimento dos sintomas, o que muitas vezes ocorre pela desinformação sobre os danos ocasionados pela exposição ^26^ ^,^ ^40^ ^,^ ^41^. 

Nota-se que, mesmo diante de transformações institucionais, tecnológicas e políticas organizadas nas últimas décadas e da inserção dos trabalhadores na APS, as práticas e a lógica de trabalho, historicamente centradas no combate ao mosquito, permanecem, em grande parte, inalteradas, preservando um caráter predominantemente operacional (combate) e não integrado em muitos municípios, apesar do avanço da legislação federal.

Na maioria das vezes, o treinamento se restringiu à transmissão oral do conhecimento e de instruções inadequadas de superiores e órgãos responsáveis pela formação ^6^. 

A situação fica exemplificada no depoimento de um trabalhador:

“*Lembrei do ‘treinamento’ que recebíamos no início da década de 90. Era massificada a frase ‘tem que jogar larvicida e inseticida’* (...) *o agente ia para o trabalho de campo com a ideia de consumir os agrotóxicos* (...) *Em alguns casos, ocorria a resistência do morador em aceitar o tratamento e muitos de nós fomos orientados a colocar uma colher de chá de Abate* [temefós], *que era o inseticida utilizado, em um copo de água e beber, para demonstrar ao morador que o produto não fazia mal*” ^6^ (p. 178).

Além da inadequação do treinamento e formação, reflexos da precarização social e do trabalho, a categoria se depara com a carência de equipamentos de proteção (coletivos e individuais). Segundo os trabalhadores, os EPI raramente são fornecidos pelos órgãos responsáveis e, de forma geral, são de má qualidade, pois não oferecem a proteção requerida e certificados de autorização ^6^ ^,^ ^8^. Ademais, o acesso não é igualitário nos municípios, fazendo com que os ACE convivam com realidades distintas, mesmo tendo as mesmas atribuições.

Ressalta-se que a utilização do EPI não impede a exposição ^42^ e o uso incorreto pode se tornar um risco ainda maior à saúde, considerando que não existe dose segura diante de substâncias neurotóxicas, carcinogênicas e para desreguladores endócrinos ^11^ ^,^ ^17^ ^,^ ^43^. Apoia essa discussão o estudo de Leme et al. ^44^, que avaliou o EPI dos profissionais do controle de endemias de São Paulo. As roupas utilizadas em campo possibilitaram a penetração de malationa, mesmo em uniformes usados pela primeira vez, constatando que trabalhadores com EPI não estavam livres da exposição ao produto químico.

No Brasil, empregados públicos e contratados são regidos pela *Consolidação das Leis do Trabalho* (CLT) e pelas *Normas Regulamentadoras* (NR’s) ^45^. Mesmo com limitações, a NR-7 estabelece as diretrizes para o desenvolvimento do Programa de Controle Médico de Saúde Ocupacional (PCMSO), com o objetivo de proteger a saúde dos trabalhadores em relação aos riscos ocupacionais. A primeira diretriz do programa aconselha a “rastreabilidade e detecção precoce dos agravos à saúde relacionados ao trabalho” e a “vigilância ativa da saúde do trabalhador,” por meio de exames médicos e coleta de informações sobre sinais e sintomas de agravos à saúde relacionados ao trabalho ^45^.

Na prática, os servidores estatutários federais e municipais, não possuíam monitoramento de saúde e, só após anos de lutas e ações judiciais por meio dos sindicatos, os exames periódicos foram iniciados em 2025 pelo Ministério da Saúde ^12^. No entanto, ainda enfrentam a falta de um valor basal do próprio trabalhador para fins de comparação ^6^ ^,^ ^43^. Essa ausência de acompanhamento inviabiliza o estabelecimento do nexo da relação entre trabalho e saúde e permite que os ACE fiquem vulneráveis às nocividades da função e das políticas de saúde vigentes.

Para além dos dados coletados e analisados, os diálogos com os ACE, por meio da CAP, permitiram ratificar o efeito fatídico da exposição à saúde desses trabalhadores. Trabalhadores estes que têm como função principal a promoção da saúde ambiental e coletiva. O diálogo permanente com os trabalhadores permitiu perceber as angústias e sofrimento vividos frente ao adoecimento e morte precoce de muitos companheiros(as) de trabalho, mas igualmente, possibilitou conhecer uma trajetória de luta por melhores condições de vida, saúde e trabalho.

## Conclusões

O estudo apresenta informações originais sobre o processo de trabalho dos ACE, caracterizando a exposição aguda e crônica a múltiplos agrotóxicos e a prevalência de sintomas de intoxicação referidos pelos trabalhadores da amostra. Evidenciou tarefas que não necessariamente envolviam aplicação de agrotóxicos por meio de bombas, mas demonstraram-se igualmente nocivas à saúde por contato e/ou manipulação deles. Além disso, foi identificada a ocorrência de diferentes agravos à saúde, inclusive à saúde mental. As exposições a agrotóxicos podem causar danos, por vezes irreversíveis e, por serem caracterizados por quadros clínicos sutis, inespecíficos e/ou comuns, fazem com que os trabalhadores se deparem com a resistência no estabelecimento do nexo desta relação, discussões que precisam avançar no campo da saúde do trabalhador, exercendo o princípio da precaução ^43^.

Ainda que se apresente como um problema de saúde pública, os casos de intoxicação ainda se encontram invisibilizados, seja nos processos de trabalho, seja nas ações de vigilância ou nas limitações dos serviços e/ou registros, pois na falha da confirmação de casos, inviabiliza as ações de proteção à saúde do trabalhador(a). Assim, é imprescindível o acompanhamento periódico e do monitoramento da saúde dos ACE, como também a mudança de paradigma no combate aos vetores, para a construção de espaços que favoreçam à saúde do(a) trabalhador(a), e isso se desdobre em mudanças no processo de trabalho.

### Limitações

A coleta de dados foi realizada por um questionário *online*, limitando-se a quem teria acesso a tecnologias digitais. Porém, tornou-se uma vantagem, pois conseguiu abranger ACE de municípios mais distantes (próximo de 10% do total trabalhadores). Para minimizar o viés de memória dos agrotóxicos utilizados, foi disponibilizada uma lista com as principais substâncias, nomes técnicos e também comerciais. Todavia, compreende-se que, ante a sua variedade, houve a possibilidade de redução dos registros, subestimando os dados. Cabe registrar o efeito do trabalhador sadio, o que pode subestimar o efeito da exposição no processo de trabalho. Por fim, por se tratar de uma amostra de conveniência, a pesquisa assume uma finalidade exploratória para avaliar a exposição dessa categoria de trabalhadores e os efeitos sobre a saúde. Entretanto, enfatizamos que, apesar das limitações do tipo de estudo com relação à amostragem, considerando o princípio da precaução ^43^, da responsabilidade dos entes governamentais (federal, estaduais e municipais), os dados da literatura sobre os efeitos dos agrotóxicos e as proibições dessas substâncias em outros países deveriam ser suficientes para que seu uso não seja central na política de combate às endemias no Brasil.

## Data Availability

Os dados de pesquisa estão disponíveis mediante solicitação à autora de correspondência.
